# Organic Salt-Doped Polymer Alloy: A New Prototype Hole Transporter for High-Photovoltaic-Performance Perovskite Solar Cells

**DOI:** 10.1021/acsami.4c19907

**Published:** 2025-02-05

**Authors:** Bing-Chen Zhang, Shang-Wen Lan, Chia-Ha Tsai, Chien-Hung Chiang, Chun-Guey Wu

**Affiliations:** Department of Chemistry, National Central University, Jhong-Li 32001, Taiwan, ROC

**Keywords:** perovskite solar cell, D-A copolymer, organic salt dopant, polymer alloy, hole transporter

## Abstract

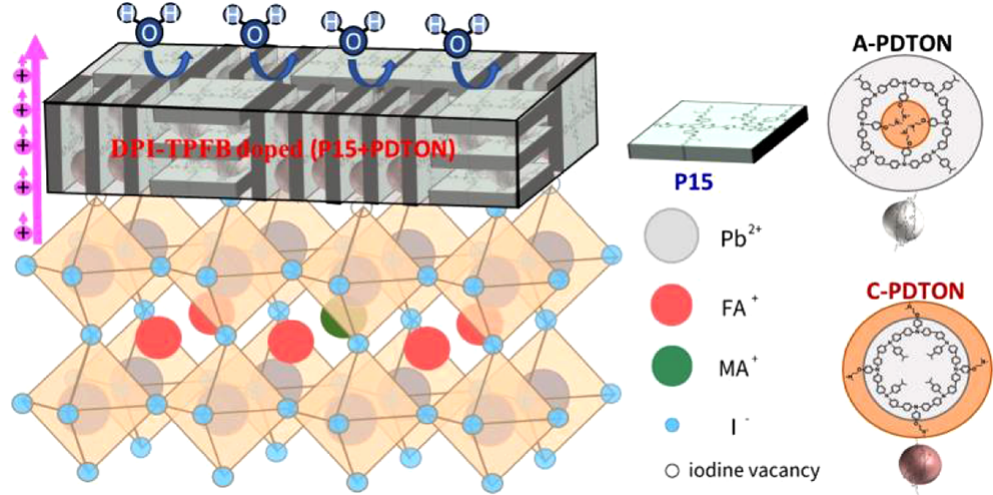

Hole-transporting layer (HTL) is one of the key components in a regular perovskite solar cell (r-PSC), which has the function of extracting the photon-excited holes from the absorber and then transporting them to the electrode. The most commonly used HTL in r-PSC is LiTFSI and tBP-doped spiro-OMeTAD. The inevitable instability induced by a deliquescent inorganic salt (LiTFSI), the migration of small lithium ions, and the necessary oxidation process in air hinder the commercialization of this technology. In this paper, a new undoped D–A copolymer **(P15)** is used as a hole-transporting material (HTM) for r-PSC but with moderate photovoltaic performance. Therefore, an organic salt, DPI-TPFB, having a big organic cation and a hydrophobic anion, was used as a dopant to increase the conductivity/hole mobility of **P15** while avoiding the instability caused by lithium salt and moisture. Furthermore, an amphiphilic polymer, PDTON (with hole- transporting and perovskite-passivation ability), was added to **P15** to form a polymer alloy, (**P15** + PDTON), to further enhance the crystallinity and, therefore, the conductivity/hole mobility of **P15** via space-confined interaction. As a result, r-PSCs based on DPI-TPFB-doped (**P15** + PDTON) HTLs exhibit the highest power conversion efficiency (PCE) of 18.8%, which is higher than those of the cells based on DPI-TPFB-doped **P15** (15.08%), DPI-TPFB-doped PDTON (7.37%), and undoped (**P15** + PDTON) (15.66%) HTLs. Cells based on DPI-TPFB-doped (**P15** + PDTON) HTL also have much better long-term stability than those using LiTFSI and tBP-doped spiro-OMeTAD as an HTL. The studies show that a polymer-compatible organic salt, DPI-TPFB, can be used as a stable dopant to increase the hole mobility of polymeric HTL without sacrificing the stability of the resulting cells, and mixing two ordinary photovoltaic performance polymeric HTLs (such as **P15** and PDTON) can form a high- photovoltaic-performance polymer alloy (**P15** + PDTON) HTL. Therefore, organic salt-doped polymer alloy can be regarded as a new prototype hole transporter for high-photovoltaic- performance PSCs.

## 1 Introduction

Perovskite solar cells (PSCs) are photovoltaic devices based on a perovskite absorber. PSCs have progressed fast since they were reported in 2009^1^ and now the certified efficiency is over 26%.^2^ Plenty of studies were carried out to improve the efficiency of PSCs by tuning the properties of the perovskite absorber^3−8^ and/or carrier transporting layers (CTLs).^9−11^ Over time, the efficiency and stability of PSCs have been increasingly limited by the CTLs.^11^ Among the CTLs, the hole-transporting layer (HTL) has the function of extracting holes from the photo-excited absorber, optimizing the HTL/perovskite interface for hole extraction, transporting holes to the electrode, forming a proper band energy alignment with the absorber, and being a substrate for depositing the electrode in a regular (p–i–n) cell architecture.^12−14^ As a result, the HTL strongly affects the energetic alignment,^15^ charge recombination kinetics,^11,16^ charge extraction efficiency,^17^ and therefore the photovoltaic performance of the cells. The dominant hole transporter used in state-of-the-art regular PSCs is p-doped spiro-OMeTAD (2,2′,7,7′-tetrakis(*N*,*N*-dipmethoxyphenylamine)-9,9′-spirobifluorene),^18,19^ which possesses a long-standing reputation as an effective solid-state HTL for dye-sensitized solar cells (DSCs)^20^ and PSCs.^21^ However, spiro-OMeTAD itself is vulnerable to thermal (>85 °C) degradation^22^ at the cell’s operational temperature, and its dopant (LiTFSI) is hygroscopic,^23^ with morphological ion migration issues.^19^ It is known that spiro-OMeTAD is a very poor barrier for the migration of small ions (stemming from its dopants) to electrodes or other PSC components^19^ to participate in undesirable side reactions^24^ that affect the long-term stability of the cells. Moreover, the necessary oxidation process of the doped spiro-OMeTAD in air also makes the cell fabrication process less controllable/reproducible. Minor variations in dopant concentration and environmental conditions (such as illumination and humidity) can also significantly influence the conductivity of doped spiro-OMeTAD, and therefore, the photovoltaic performance of the corresponding cell.^25^ As a result, searching for good HTLs beyond doped spiro-OMeTAD is one of the hot research topics. A lot of small organic molecules^26−31^ and polymers^32−35^ have been proven to be excellent hole-transporting materials (HTMs) for high-efficiency and stable PSCs. Furthermore, some organic hole-transporting materials may be also the passivators for the perovskite absorber,^32,36,37^ further improving the efficiency and stability of the corresponding cells. Considering the multiple functions of HTLs, the fundamental requirements, such as high hole mobility and stability, good solubility and hydrophobicity, proper film morphology and frontier orbital energy level, passivation ability, and stable dopants (if doping is needed), making an exquisite design of HTLs is necessary. Polymeric HTMs, having the advantages of good film morphology and high thermal stability, are among the best choices for PSCs.

**Figure 1:**
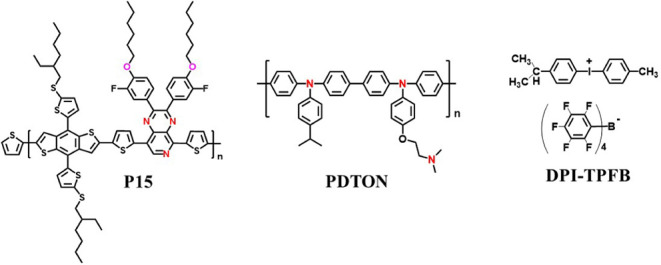
Chemical structures of P15, PDTON, and DPI-TPFB.

Most of the highly efficient polymer HTMs for PSCs are donor (D)–acceptor (A) copolymers,^32^ since the alternation of donor and acceptor moieties is one of the most important characteristics for the hole to transport in the polymer backbone. Another critical point to obtain very efficient polymer HTLs is the close contact between polymer chains, since conductivity is normally related to charge flow along the conjugated backbone as well as hopping between polymer chains.^38−47^ The interaction between the donor and acceptor units (from two different polymer chains) may help the polymer chains come close. Another advantage of D–A copolymers is that their HOMO/LUMO energy levels can be tuned easily by the D and A units to fit the perovskite absorbers with various compositions and frontier orbital energy. PCBTDPP, made from carbazole, thiophene, and 2,5-dihydropyrrolo-[3,4]pyrrole-1,4-dione (DPP) units, is the first reported dopant-free D–A copolymer HTM used in a perovskite solar cell, but with a limited power conversion efficiency of only 5.55%.^41^ Furthermore, the benzodithiophene (BDT) unit, an inherent electron donor group that can be inserted into other organic moieties with simple synthetic approaches, is also one of the most promising donors used in dopant-free polymer HTLs.^41^ BDT units were also found in the backbone of the famous high-photovoltaic-performance p-type polymers (PTB7 and PTB7-th) for the bulk heterojunction (BHJ) polymer solar cells.^42^ For the acceptor part, a quinoxaline derivative with two electron-withdrawing imines to tune the optoelectronic properties of the resulting polymer was chosen.^43^ By further incorporating pyridine substitution into the quinoxaline unit, it could an additional opportunity to alter the electronegativity of quinoxaline derivatives to obtain a polymer with a low valence band level. The resulting D–A copolymer (PBDT-TPQ) was first designed to be used as a p-type polymer in BHJ polymer solar cells, and the highest efficiency of 4.4% was achieved.^44^ To further improve the photovoltaic performance of (PBDT-TPQ), the alkoxyl substituent in PBDT-TPQ was replaced with 2-ethylhexyl)thio)thiophene, and fluoride was added to the phenyl of TPQ to increase the interaction between polymer chains. The resulting D–A copolymer (poly-{8-(5-(4,8-bis(5-((2-ethylhexyl)thio)thio-phen-2-yl)-6-(thiophen-2-yl)benzo[1,2-b:4,5-b’]dithiophen-2-yl)thiophen-2-yl)-2,3-bis(3-fluoro-4-(hexyloxy)phenyl)-5-(thiophen-2-yl)pyrido[3,4-*b*]pyrazine) (**P15,** its structure is displayed in Figure 1) has a low photovoltaic performance of 4.5% when it was applied as a p-type polymer in a BHJ polymer solar cell.^44^ However, based on the physicochemical properties/functionality of **P15** that we had found, **P15** could be a suitable HTM for PSC, besides its hole mobility is lower than that of LiTFSI and tBP-doped spiro-OMeTAD.

To enhance the hole mobility of **P15** while avoiding the instability caused by the inorganic-based dopant, two strategies (using organic salt as a dopant and forming a polymer alloy) were used in this study. To avoid the instability caused by the inorganic lithium salt dopant, a polymer-compatible salt, DPI-TPFB (4-isopropyl-4′-methyldiphenyliodonium)tetrakis (penta-fluorophenyl)borate^48^ with a large organic cation and a hydrophobic anion (its structure is also shown in Figure 1), was used to dope **P15**. DPI-TPFB is a proton dopant that can protonate the basic pyrido[3,4-*b*]pyrazine moiety in **P15** to increase its hole mobility.^49^ Moreover, a polymer alloy can be defined as two or more polymers having a physical or chemical interaction when they are blended together. It can be regarded as a new material with a single phase but differing in physical properties from its individual components.^50^ In other words, a polymer alloy may have different energy levels and mobilities compared to its individual polymers. Polymer alloy has been used to enhance the photovoltaic performance of polymer solar cells several years ago.^51^ Recently, Liu et al. reported that a PM6 + PMSe polymer alloy can achieve higher power conversion efficiency (PCE) than individual PM6 or PMSe alone when it is used as the HTM for regular perovskite solar cells.^37^ In this study, an amphiphilic polymer, PDTON (its structure is also displayed in Figure 1), which was shown to be a hole-transporting material with passivation ability toward perovskite,^52^ was added to **P15** to form a polymer alloy (**P15** + PDTON) to increase the hole mobility of **P15**. Regular perovskite solar cells based on DPI-TPFB-doped (**P15** + PDTON) polymer alloy HTL achieved the highest efficiency, close to 19%, and have much better long-term stability compared to the cells based on LiTFSI and tBP-doped spiro-OMeTAD HTL. Table S1 displays some HTLs using DPI-TPFB or LiTFSI and tBP as dopants and their photovoltaic performance.

## 2 Results and Discussion

The scheme for the synthesis of the D–A copolymer **P15** is illustrated in Scheme S1. **P15** was synthesized by Stille coupling of the corresponding donor and acceptor. The detailed synthetic steps of the intermediates are the same as what we reported previously,^45,46^ except the starting materials were slightly different. The structures of the donor and acceptor, as well as the final copolymer, were identified with ^1^H NMR as displayed in Figure S1. The molecular weight of **P15** was determined with GPC (Gel Permeation Chromatography) using polystyrene as the standard and TGA analysis is displayed in Figure S2a,b respectively. The molecular weight of **P15** is close to 30K and stable up to 250 °C. The photovoltaic parameters of regular perovskite cells (using a cell fabrication SOP developed in our lab) based on **P15** (both nondoped and doped) and LiTFSI, tBP-doped spiro-OMeTAD HTLs^53^ are listed in Table S2. The data clearly suggested that organic salt DPI-TPFB is a better dopant for **P15** compared to the inorganic dopant LiTFSI. Data listed in Table S2 also reveal that regular PSCs based on DPI-TPFB-doped **P15** HTL (compared to the cell based on LiTFSI, tBP-doped spiro-OMeTAD HTL) have two obvious weaknesses: the PCE is too low and the standard deviation of the efficiency is too high. The low efficiency may be due to the incompatibility between perovskite and **P15**, and the high deviation in efficiency may come from the inhomogeneity of the **P15** film on perovskite (also related to the incompatibility of the Psk/**P15** interface). To improve the photovoltaic performance of the cell based on DPI-TPFB-doped **P15** HTL, two strategies: perovskite/P15 interface modification and changing the properties of **P15** by forming a polymer alloy, are used in this study.

It was well-known that the interface modification between the perovskite absorber and the carrier transporting layer (CTL) can improve the efficiency and long-term stability of perovskite solar cells.^47−49,54−62^ The functions of the interface modification agent include passivating the perovskite defects at the interface, promoting the carrier extraction of the CTL, aligning the frontier orbitals’ energy levels, and improving the compatibility between the perovskite (Psk) and the CTL, among others. Lots of molecules can be used as interface modification agents, such as Lewis bases, organic salts, carbon materials, inorganic solid-state materials, oxides, quantum dots, organic polymers, as well as perovskite materials.^54−62^ In this study, an amphiphilic polymer named PDTON (poly(isopropylethoxyamino)triphenylamine)^52^ was chosen as Psk/**P15** interface modification agent as well as a polymer for alloying with **P15**. PDTON has a triphenylamine backbone and an alternative hydrophobic alkyl chain and hydrophilic alkylamine at the side chains, which can interact with the conjugated **P15** HTL (via the alkyl chain) and the ionic perovskite absorber (via the amino group) simultaneously. Its triphenylamine backbone, having the hole-transporting ability,^63^ can act also as a cohole-transporting layer (Co-HTL) in PSCs. After optimizing the Psk/**P15** interface with PDTON films, a cell with smaller current hysteresis was obtained, but the efficiency and efficiency deviation improved insignificantly, as shown in the photovoltaic parameters listed in Table 1. Therefore, as mentioned in the previous paragraph, Liu^37^ et al. had used two polymers to make a polymer alloy HTL for regular perovskite solar cells to achieve higher PCE compared to those based on a single polymer HTL. Therefore, PDTON was added to **P15** to form a (**P15** + PDTON) polymer blend (or alloy) HTL, and the corresponding PSCs achieved the highest PCE of 18.82%, as shown in the photovoltaic data also listed in Table 1. The photovoltaic parameters of the cells based on PDTON (both undoped and DPI-TPFB-doped), DPI-TPFB-doped **P15,** and DPI-TPFB-doped (**P1**5 + PDTON) polymer alloy (or blend), as well as LiTFSI, tBP-doped spiro-OMeTAD, were also collected in Table 1 for comparison. *I*–*V* curves of the cells are displayed in Figure S3 and

**Table 1:** Photovoltaic Parameters of Regular Perovskite Solar Cells Based on Seven HTLs^a^^b^^c^^d^^e^

HTL	*J*_sc_ (mA/cm^2^)	^a^Inte. *J*_sc_ (mA/cm^2^)	Voc (V)	FF (%)	PCE (%)	^b^HI
Undoped P15	22.96^c^ (22.31 ± 0.37)	---	1.03 (1.02 ± 0.01)	47 (47 ± 1)	11.08 (10.73 ± 0.29)	---
^d^Doped P15	24.6 (24.17 ± 0.4)	20	1.03 (1.03 ± 0.01)	60 (54 ± 3)	15.08 (13.57 ± 1.03)	20%
PDTON/doped P15	23.8 (23.41 ± 0.42)	20	1.03 (1.03 ± 0.01)	65 (60 ± 3)	15.92 (14.5 ± 0.86)	9.3%
Undoped PDTON	20.97 (19.13 ± 1.83)	---	0.87 (0.84 ± 0.03)	37 (36 ± 1)	6.81 (5.82 ± 0.9)	---
Doped PDTON	20.42 (19.66 ± 0.64)	---	0.92 (0.89 ± 0.03)	39 (39 ± 1)	7.37 (6.76 ± 0.52)	---
Undoped (P15 + PDTON)	22.88 (22.68 ± 0.14)	---	1.06 (1.04 ± 0.02)	65 (63 ± 2)	15.66 (14.87 ± 0.9)	---
Doped^e^ (P15 + PDTON)	24.29 (24.26 ± 0.52)	21	1.08 (1.06 ± 0.01)	72 (70 ± 1)	18.82 (18.09 ± 0.38)	1.6%
Doped spiro-OMeTAD	25.3 (24.5 ± 0.7)	---	1.13 (1.13 ± 0.02)	73 (73 ± 2)	20.9 (20.01 ± 0.59)	---
PDTON/Doped spiro-OMeTAD	24.8 (24.24 ± 0.66)	20	1.17 (1.17 ± 0.02)	76 (72 ± 2)	21.94 (20.52 ± 0.82)	2.9%

a The current density integrated from the IPCE curves.

b HI = (PCE_reverse_ – PCE_forward_)/PCE_reverse_ × 100%.

c The average of 16 cells.

d Dopant(s) for P15 is DPI-TPFB, for spiro-OMeTAD, are Li-TFSI and tBP.

e The weight ratio of P15:PDTON = 9:1.

the distribution of the photovoltaic parameters of the cells is illustrated in Figure S4 (the *I*–*V* curves and the distribution of the photovoltaic parameters for the low PCE cells were omitted in Figures S3 and S4 to show the difference between HTLs more clearly). Data shown in Table 1 reveal that PDTON (both doped and undoped) can be an HTL for regular perovskite solar cells but with low photovoltaic performance. PDTON can also be a Psk/doped **P15** interface modification agent to slightly improve the efficiency and standard deviation of the corresponding solar cells, probably through improving the compatibility between Psk and **P15** or the quality (homogeneity and conductivity) of the doped **P15** film deposited on PDTON. Nevertheless, when PDTON was blended with **P15** to form a polymer blend (or alloy) and doped with DPI-TPFB, the efficiency and the homogeneity/reproducibility of the corresponding cells improved significantly (the standard deviation of the photovoltaic parameters is related mostly to the film homogeneity and reproducibility of the HTL for the regular perovskite solar cells in this study). To focus more on the benefits of using an organic dopant and the (**P15** + PDTON) polymer blend (or alloy) HTL to enhance the photovoltaic performance of regular perovskite solar cells, only four regular PSCs based on DPI-TPFB-doped **P15** (named **P15**) and DPI-TPFB-doped (**P15** + PDTON) (named (**P15** + PDTON)), as well as PDTON used as Psk/**P15** and Psk/LiTFSI, tBP-doped spiro-OMeTAD (named PDTON/**P15** and PDTON/spiro-OMeTAD, respectively) interface modification agents, were studied in detail in the following sections.

**Figure 2:**
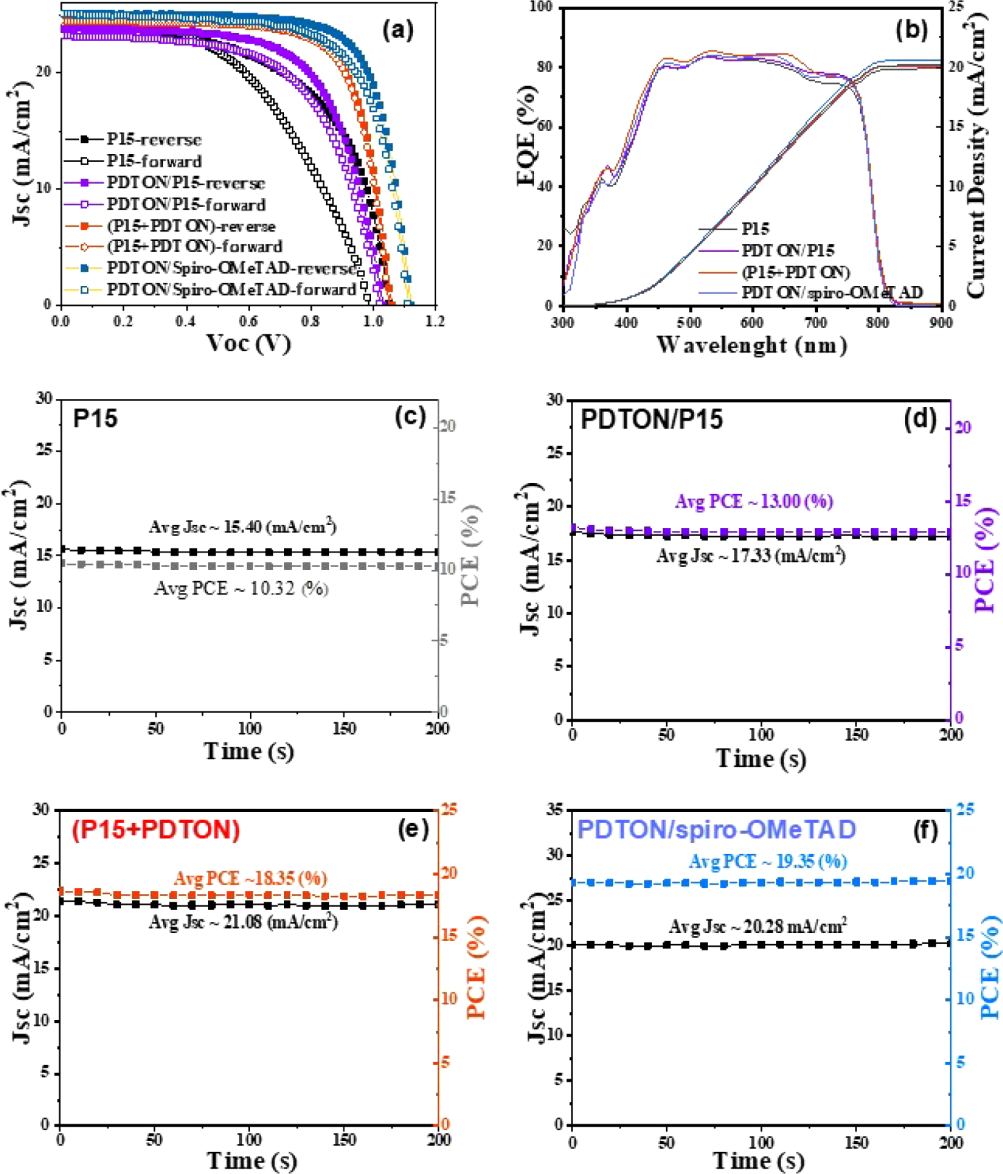
(a) *I–V* curves (both forward and reverse scans) and (b) IPCE curves for the cells based on four HTLs. (c–f) Steady-state power output of the cells based on four HTLs.

**Figure 3:**
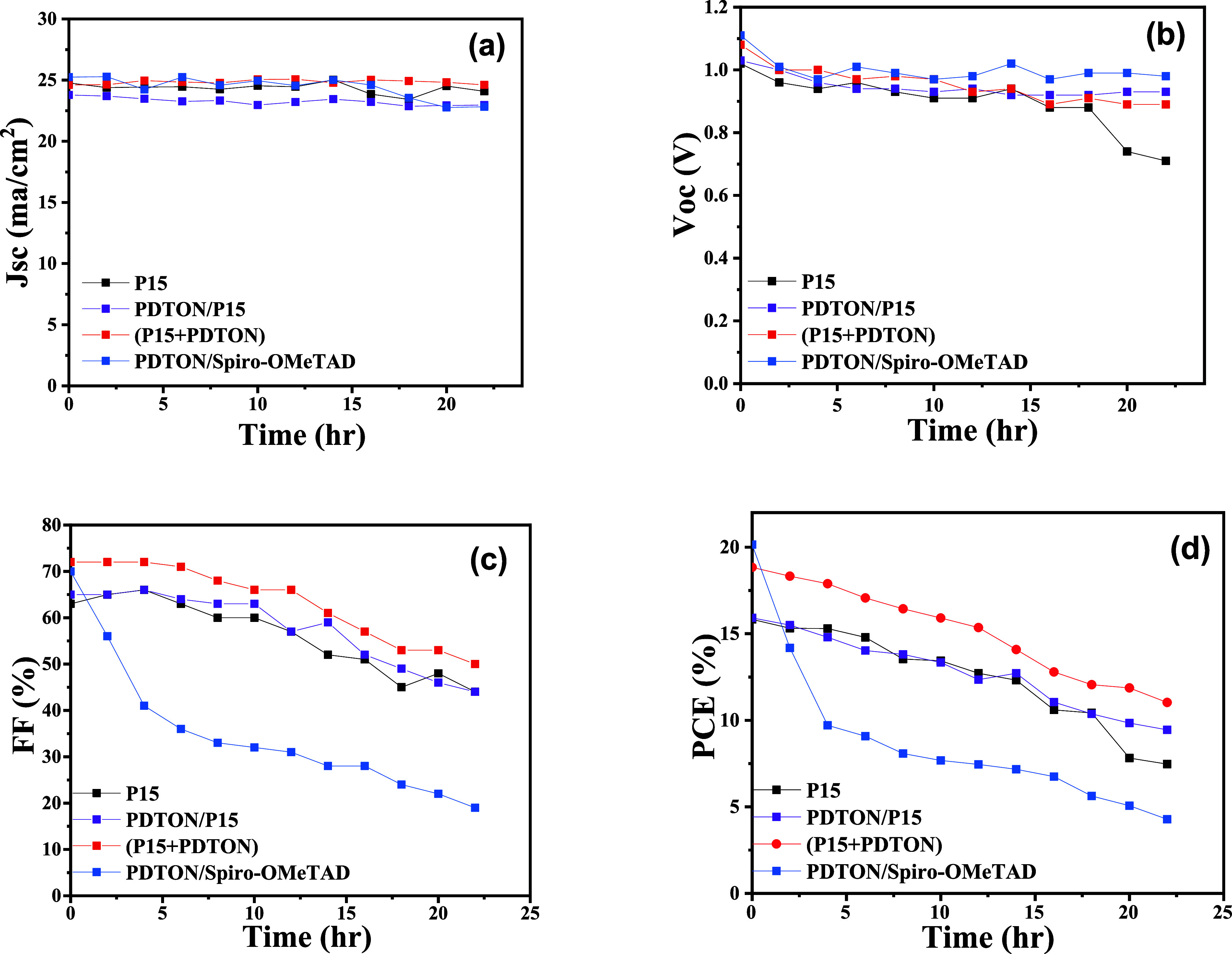
Long-term stability (including four photovoltaic parameters) of the cells based on four HTLs: (a) *J*_sc_ values, (b) Voc values, (c) fill factor, and (d) power conversion efficiency.

The *I–V* curves at forward and backward scans, as well as the IPCE curves of the four regular cells, are displayed in Figure 2a,b (the *J*_sc_ integrated from the IPCE curves and current hysteresis index (HI) are also listed in Table 1). Cells based on (**P15** + PDTON) HTL have not only higher efficiency but also much lower current hysteresis (HI) than those based on **P15** HTL. A negligible current hysteresis was detected for the cells based on (**P15** + PDTON) HTL, but a very high hysteresis index of ∼20% was found for cells based on **P15** HTL. The efficiency of cells based (**P15** + PDTON) HTL is lower than that of cells using PDTON/spiro-OMeTAD as an HTL; however, the HI value is smaller. The HI value will affect the long-term stability of the cell since the value is related to the imbalance of carrier transport or nonradiative recombination/accumulation close to the Psk/CTL interface.^64−67^ The hysteresis problem is alleviated by the insertion of a layer of PDTON between **P15** and Psk, although the cell based on PDTON/**P15** HTL still has an HI value of 9.3%. The IPCE curves of all four cells show a nonzero spectral response covering the UV to visible region (300–800 nm), appearing as a typical IPCE curve for lead-based perovskite solar cells. The integrated *J*_sc_ (taken from the EQE spectrum under 1 sun (AM 1.5 G) illumination) is 19.89, 20.13, 20.64, and 20.12 mA/cm^2^ for the cells based on **P15**, PDTON/**P15**, (**P15** + PDTON), and PDTON/spiro-OMeTAD HTLs, respectively. The deviation in current density integrated from the IPCE curves and *J*_sc_ values obtained from the *I*–*V* measurement for the cells based on the four HTLs did not show a significant difference. Steady-state output power, which mimics the real working condition of a solar cell under a bias at *P*_max_, is more reliable data for the authentic performance of a solar cell. The steady-state power output of the cells based on the four HTLs is shown in Figure 2c–f. For four cells, the output efficiency and current density were very stable for at least 200 s under continuous 1 sun (AM 1.5 G) illumination in the glove box at the bias voltage of *P*_max_. Nevertheless, the differences between the PCE at *P*_max_ and those calculated from the *I–V* curves for the cells based on the four HTLs are not the same. PSC

based on (**P15** + PDTON) HTL has only a 0.5% difference in efficiency, while the efficiency differences for the cells based on **P15**, PDTON/**P15,** and PDTON/spiro-OMeTAD are 4.9%, 2.9%, and 2.6%, respectively. The steady-state power output data revealed that the cell based on (**P15** + PDTON) HTL has the power output most closely aligned to the PCEs (calculated from the *I*–*V* curves) indicated. The long-term stability of the cells (Figure 3) was tested by putting them in an ambient atmosphere (30 °C, 20% relative humidity) without packing and measuring (in air) the changes of the photovoltaic parameters every 2 h. Without packing, the cell based on (**P15** + PDTON) HTL has similar stability compared to that using PDTON/**P15** as an HTL but is much more stable than the cell based on PDTON/spiro-OMeTAD HTL. It seems that the stability of the cells depends mainly on the dopant. It is reasonable to explain that the stability is related to the fact that the big cation of the organic salt DPI-TPFB migrates more difficultly compared to the small Li^+^ ion, and DPI-TPFB is less hydrophilic than the inorganic salt Li-TFSI. The big 4-isopropyl-4′-methyldiphenyliodonium cation is also too big to insert into the framework of the perovskite lattice, which can avoid damage to the Psk framework by the dopant. The hydrophilicity of the HTLs was proved using water contact angle data shown in Figure S5. Ionic perovskite is a hydrophilic material with a water contact angle of 44.8°, and the amphiphilic PDTON film (prepared by spin coating from a trichlorobenzene solution) has a contact angle of 82.5°. The DPI-TPFB-doped **P15** film is hydrophobic, having a large water contact angle of 101.5°. The DPI-TPFB-doped **P15** film deposited on PDTON has a contact angle of 102.3°, which is very similar to that coated on the Psk film, suggesting that the PDTON interface modification layer did not significantly affect the hydrophilicity of **P15** deposited on it. The water contact angle of the DPI-TPFB-doped (**P15** + PDTON) film is 92.1° (slightly hydrophobic), which is in between the contact angles of PDTON and doped **P15** but higher than that (73.4°) of the Li-TFSI, tBP-doped spiro-OMeTAD film (which is the most hydrophilic among the four HTLs well-studied in this paper). The water contact angle data suggested that the long-term stability of regular PSCs may also be related partially to the hydrophilicity of their HTLs. The stability of the cells under AM 1.5 G (1 sun) illumination is also tested, which is very close to that under room light lighting in the first 10 h; therefore, we did not study further. When the cells without packing were heated at 85 °C for 2 h, the efficiency of the cell using Li-TFSI and tBP-doped spiro-OMeTAD HTL dropped to zero, and only 30% of the original efficiency left for the cell based on DPI-TPFB-doped (P15 + PDTON) HTL. These results also reveal that the thermal stability of the cells was also improved by the organic dopant. The next questions are whether (**P15** + PDTON) is a polymer alloy or just a polymer blend and how PDTON enhances the photovoltaic performance of our newly developed **P15**, and therefore the efficiency of the corresponding regular PSCs?

**Figure 4:**
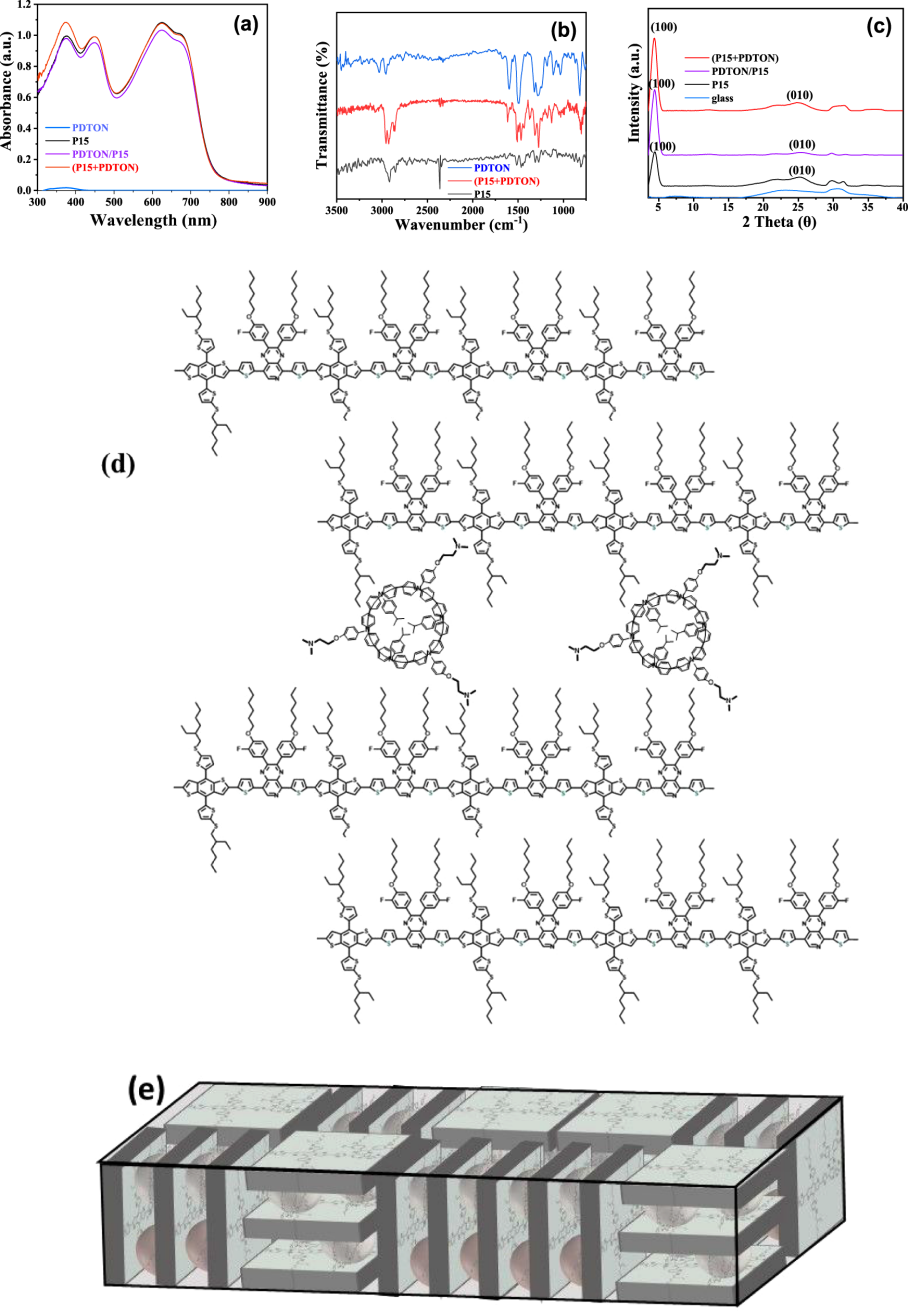
(a) UV/vis/NIR absorption spectra, (b) IR spectra, and (c) XRD diffraction patterns of P15, PDTON/P15, and (P15 + PDTON) films. (d) The interaction between P15 and PDTON in (P15 + PDTON). (e) Schematic representation of the HTL structure in (d).

Polymer alloy (made from two or more polymers) can be regarded as a new material with physical properties (such as energy levels, mobility, film morphology, polymer chain stacking, compatibility with electrode and perovskite absorber, etc., which may influence the photovoltaic performance of the corresponding cell, different from its individual components.^37,51^ Therefore, the first experiment is to verify the property difference between **P15**, PDTON, and (**P15** + PDTON). UV/vis/NIR absorption spectra of **P15**, PDTON, and (**P15**+PDTON) are illustrated in Figure 4a. The characteristic absorption peaks at 370, 451, and 625 nm of **P15** did not shift, and the ratio of the peak intensity at 451 and 625 nm (PDTON has the absorption at 370 nm) for **P15** and (**P15** + PDTON) is the same. Furthermore, infrared (IR) spectra for **P15**, PDTON, and (**P15** + PDTON) displayed in Figure 4b also show no shift of the absorption peaks of **P15** when it was mixed with PDTON. Electronic and vibrational data seem to suggest that mixing **P15** and PDTON may just form a simple polymer blend instead of a polymer alloy. Nevertheless, GIXRD patterns of **P15, P15** on PDTON, and (**P15** + PDTON), displayed in Figure 4c, show that the intensities of the diffraction peaks are different. Two diffraction peaks at 2θ of 4.4° and 25°, corresponding to the lamellar stacking and π–π stacking of **P15** chains, were observed in all the three samples. When **P15** was coated on PDTON, the crystallinity of lamellar stacking increased, but the π–π stacking slightly decreased, suggesting that **P15** and PDTON have some interaction. Furthermore, the peak intensity at 4.4° (lamellar stacking) of (**P15** + PDTON) film is 2 times higher than that of the **P15** film, but the peak intensity at 25° (π–π stacking) for both samples is almost the same. GIXRD data suggested that adding PDTON in **P15** increases the crystallinity of the **P15** film, although this change was not observed in electronic (UV/vis/NIR) and vibrational (IR) spectra. Therefore, we can say that **P15** forms a polymer alloy with PDTON since the crystallinity of **P15** in pure **P15** and (**P15** + PDTON) is not the same. The mechanism for PDTON to enhance the crystallinity of **P15** may be through space confinement as illustrated in Figure 4d,e.

**Figure 5:**
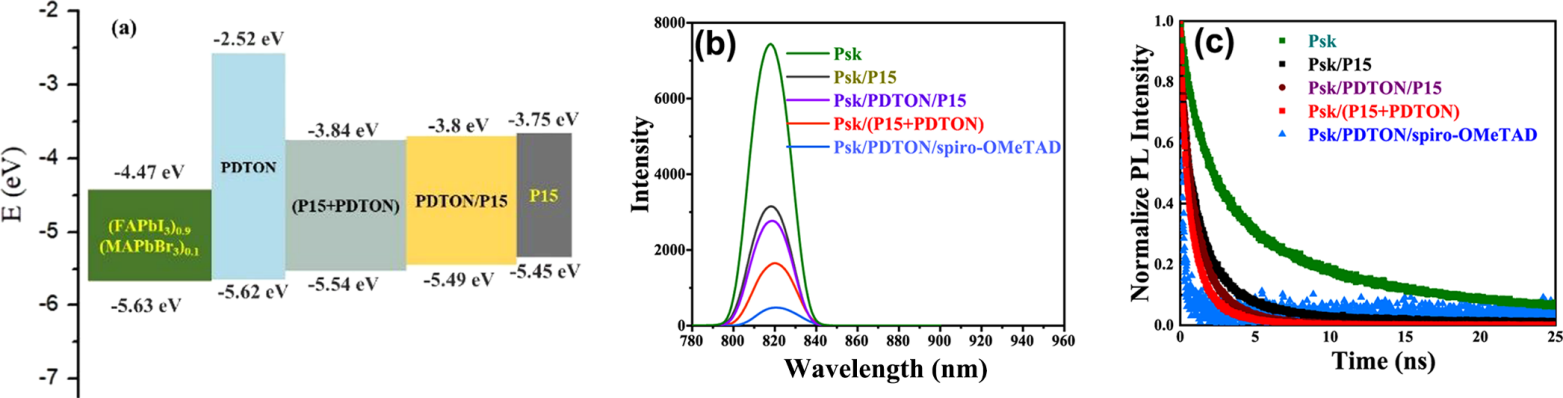
(a) Frontier orbital energy levels of four HTLs and Psk. (b) PL and (c) TRPL curves of Psk and Psk coated with four HTLs.

To be a good HTL for PSC, the frontier orbital energy level should align well with the perovskite absorber to reduce the voltage loss during hole transporting. It is interesting to know the change in the frontier orbital energy level of **P15** when it was mixed with PDTON. The frontier orbital energies (estimated from the Tauc plots of the absorption spectra and UPS spectra^68,69^ displayed in Figure S6) for **P15**, **P15** on PDTON, and (**P15** + PDTON), as well as the perovskite film used in this study, are shown in Figure 5a. It seems that adding PDTON did not meaningfully affect the band gap energy of the **P15** films. However, adding PDTON with a lower HOMO potential does slightly raise the HOMO

level of **P15** to be matched better with Psk prepared in this study. Moreover, it is intuitive to sense that increasing the crystallinity of a material will enhance its carrier mobility. There are two general methods used to reveal the mobility of a hole-transporting layer. One method is to calculate the hole mobility directly from the *I*–*V* curve of the hole-only device using SCLC (space–charge–limited current) theory.^70,71^ The other method is to measure the steady-state photoluminescence (ss-PL) and time-resolved photoluminescence (TRPL) of HTL deposited on the perovskite film to extract the related efficiency in the hole extraction/movement.^72−76^ The hole mobility of the four HTLs calculated from the *I–V* curves (displayed in Figure S7) of the hole-only devices based on SCLC theory is listed under the *I–V* curves. The hole mobilities of the **P15**, PDTON/**P15**, (**P15** + PDTON), and PDTON/spiro-OMeTAD are 1.36 × 10^–4^, 2.01 × 10^–4^, 3.39 × 10^–4^, and 3.66 × 10^–4^ cm^2^ V^–1^·s^–1^, respectively. The hole mobility of **P15** can be enhanced by increasing the crystallinity through forming a polymer alloy with PDTON. This could be one of the reasons that PSC based on (**P15** + PDTON) HTL exhibits higher PCE, Voc, and is more reproducible than that using **P15** as an HTL.

The PL spectra of Psk coated with **P15**, PDTON then **P1**5, (**P15** + PDTON), or PDTON then spiro-OMeTAD, as well as the Psk film, are displayed in Figure 5b. It is known that the PL intensity of Psk/CTL is related to the carrier extraction ability/mobility of CTL as well as nonradiative recombination losses at the Psk/CTL interface: lower PL intensity means higher carrier extraction ability and mobility of CTL or more significant nonradiative recombination losses at the interface. PL intensity for the five samples is in the order of FTO/Psk ≫ FTO/Psk/**P15** > FTO/Psk/PDTON/**P15** > FTO/Psk/(**P1**5 + PDTON) > FTO/Psk/PDTON/spiro-OMeTAD. The PL data indicated (**P15** + PDTON) has better hole-extraction ability/mobility compared to **P15** and PDTON/**P15** but worse than PDTON/spiro-OMeTAD. Lower PL intensity may also suggest that the nonradiative recombination losses at the Psk/spiro-OMeTAD interface are more significant compared to the other samples. TRPL measurements of neat Psk film and Psk covered with various HTLs were also carried out to see the charge transport kinetics, and the curves are illustrated in Figure 5c. Two carrier lifetimes calculated from the TRPL curves are collected in Table S2. Figure 4b clearly shows that the PL decay pattern for neat Psk is different from Psk with HTL overlay, attributed to the rapid extraction of charges by HTL or introduced an interface nonradiative recombination loss. Hou^77^ et al. suggested that the rapid initial decay is due to the loss of charge carriers at the interfaces, which is primarily attributed to charge extraction toward the transport layers (τ1) and subsequent interface recombination (τ2). The PL decay for Psk/HTL is dominated by hole extraction of the HTL used in this study. Psk/spiro-OMeTAD has the shortest τ1 value, suggesting that spiro-OMeTAD is the fastest hole extractor; however, it has the disadvantage of poor stability. The order of the average carrier lifetime is also parallel to the PL data, also indicating that (**P15** + PDTON) has better hole extraction and transporting ability than **P15** and PDTON/**P15** but worse than PDTON/spiro-OMeTAD. Both ss-PL and TRPL data suggest that adding PDTON to P15 can improve the hole extraction and transporting ability of **P15**, probably through increasing the crystallinity and hole mobility of **P15** by forming a polymer alloy. Consistent with the photovoltaic data listed in Table 1 and Figure 3, a cell based on LiTFSI and tBP-doped spiro-OMeTAD HTL has high efficiency but poor long-term stability. On the other hand, the combination of a polymer alloy with an organic dopant, a new HTL with efficiency close to that of Li-TFSI and tBP-doped spiro-OMeTAD but having better stability, can be realized.

**Figure 6:**
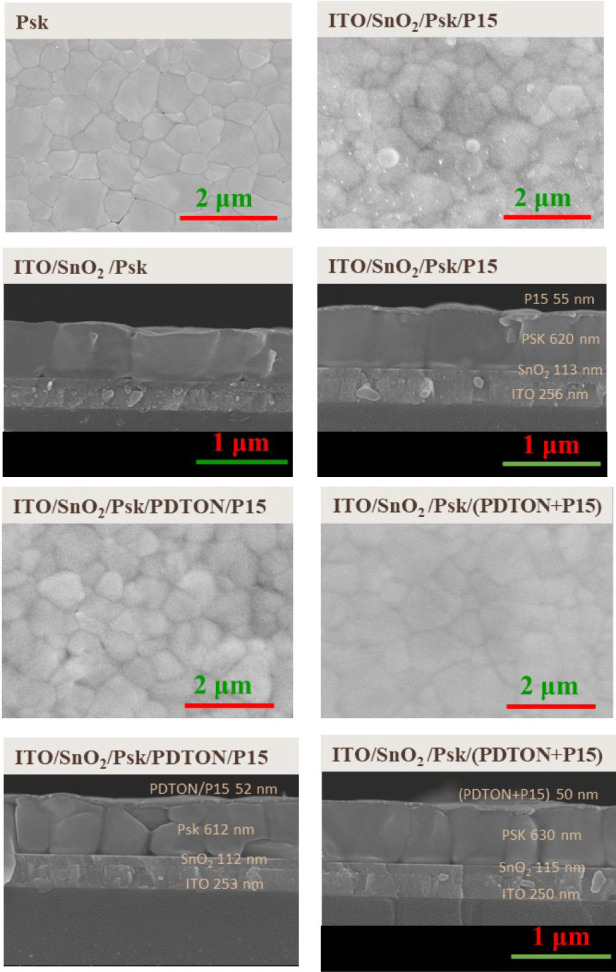
SEM topography and cross-sectional images of Psk film and Psk coated with HTL.

The luminescence intensity and carrier lifetime of Psk coated with HTL depend on the hole extraction and transporting ability of the HTL, as well as the Psk/HTL interface nonradiative recombination loss. SEM topography and cross-section images of Psk film, as well as Psk coated with HTL, are illustrated in Figure 6. The surface morphology of the Psk film and (**P15** + PDTON) is very flat, and the surface roughness of **P15** and PDTON/**P15** is higher than that of (**P15** + PDTON). The bright spot on the **P15** film is due to charging, probably because of its low conductivity. The SEM cross-section images did not show an observable difference under 100,000 times magnification. Therefore, the better photovoltaic performance of (**P15** + PDTON) HTL compared to **P15** (or PDTON/**P15**) is most probably due to (**P15** + PDTON) having higher hole mobility, which is attributed to the increase in **P15** crystallinity by forming an alloy with PDTON.

## 3 Conclusion

In this paper, a new copolymer (**P15**) was prepared to be used as an HTM (replacing the unstable LiTFSI, tBP-doped spiro-OMeTAD HTL) for regular perovskite solar cells (PSCs) to improve cell stability. Nevertheless, the efficiency of PSCs based on pristine (undoped) **P15** HTL is much lower than the state-of-the-art PSCs using doped spiro-OMeTAD as an HTL. Therefore, a polymer-compatible salt (DPI-TPFB) with a big organic cation was used as a dopant for **P15** to increase its conductivity and avoid the disadvantages of the hydrophilic LiTFSI and tBP dopants used in spiro-OMeTAD. Furthermore, an amphiphilic polymer, PDTON, was added to **P15** to form a polymer alloy (**P15** + PDTON) to enhance the hole mobility further (therefore the photovoltaic performance) of the **P15**-based HTL via increasing the crystallinity of the **P15** polymer chains. As a result, regular perovskite solar cells based on DPI-TPFB-doped (**P15** + PDTON) HTL exhibit the highest efficiency of 18.8% with small current hysteresis and are very reproducible. Although the PCE of PSCs based on DPI-TPFB-doped (**P15** + PDTON) HTL is lower, the stability is much better than that of cells based on LiTFSI, tBP-doped spiro-OMeTAD HTL. The DPI-TPFB dopant creates a hydrophobic HTL surface, and its big organic cation can reduce ion movement to improve the stability of the corresponding cell. The function of PDTON in the polymer alloy is, on the other hand, to increase the order of **P15** chains through space confinement to enhance the hole extraction and mobility of **the P15** based HTL. In this aspect, organic salt-doped polymer alloy can be regarded as a new prototype hole transporter for high-photovoltaic-performance PSCs. These results reveal an exciting fact that there exist huge opportunities to find excellent polymeric HTMs (such as polymer alloys, even doped) to enhance simultaneously the efficiency, long-term stability, and reproducibility of perovskite solar cells.

## 4 Experimental Section

### 4.1 Chemicals and Physicochemical Studies

Five nm SnO_2_ water suspension (15 wt %) was acquired from Alfa Aesar, USA, and then concentrated to 20 wt % as a stock solution for the preparation of ETLs. FTO/glass (TEC-7) was obtained from MTI, USA, and patterned in our lab. Lead iodide, lead bromide (PbI_2_, PbBr_2_; Alfa Aesar, USA), methylammonium bromide (MABr), formamidinium iodide (FAI), bis((trifluoromethane)-sulfonimide lithium salt (Li-TFSI, 99%), 4-*tert*-butylpyridine (tBP), and 4-isopropyl-4′-methyldiphenyliodonium tetrakis(pentafluorophenyl)borate (DPI-TPFB) were purchased from Ossila Co., UK. 2,2′,7,7′-Tetrakis(*N*,*N*-di-*p*-methoxyphenylamino)-9,9′-spirobifluorene (spiro-MeOTAD, Luminescence Technology, Taiwan) and other compounds for the synthesis of the copolymers used in this study were purchased from commercial sources. All chemicals, unless specified, were used as received without further purification.

GIXRD data were collected in the 2θ range of 5–40 degrees on a Bruker powder diffractometer (D8 Discover) using Cu Kα1 radiation equipped with a 2D detector. UPS and XPS spectra were obtained with a Thermo VG-Scientific/VG-Sigma Probe spectrometer. UV/vis/NIR and PL spectra were recorded by a Cary 300 Bio spectrometer and a Hitachi F-7000 fluorescence spectrophotometer, respectively, at room temperature. The nanosecond time-resolved photoluminescence (TRPL) was performed with an optical-microscope-based system (UniRAM, Protrustech) with a custom-designed light path. The average power, wavelength, pulse duration, and repetition rate of the excitation are 20 μW, 405 nm, 150 ps, and 20 MHz, respectively. Scanning electron microscopy (SEM) was performed with a Hitachi S-800 microscope at 15 kV. Samples for SEM imaging were mounted on a metal stub with a piece of conducting tape, then coated with a thin layer of gold film to avoid charging. Contact angles were measured with a homemade setup (Grandhand Ctag01, Taiwan) using water as a probe. The contact angle is determined by using the image of a sessile drop at the points of intersection between the drop contour and the projection of the surface.

### 4.2 Preparation of the Hole-Transporting Layers (HTLs) for Regular Perovskite Solar Cells (PSCs)

In this study, D–A copolymers (**P15**) and polymer alloy (**P15** + PDTON), as well as spiro-OMeTAD, were used as HTLs in regular PSCs. The method for the synthesis of **P15** is similar to what we reported previously,^44,45^ except different starting materials were used, and the synthetic scheme is illustrated in Scheme S1, SI. The structure of **P15** was identified with ^1^H NMR spectra. PDTON was prepared according to the literature and characterized with ^1^H NMR.^52^ DPI-TPFB doping was performed by adding 0.5 mg (10 wt % vs copolymer) dopant into 1.5 wt % **P15** (or (**P15** + PDTON) with various **P15**:PDTON ratios, the best weight ratio is 9:1) solution (trichlorobenzene is the solvent). The DPI-TPFB-doped **P15** (or (**P15** + PDTON)) solution was irradiated under the UV lamp (365 nm, 16 W) for 5 min, then spin-coated (4500 rpm for 30 s) on top of the perovskite film to be an HTL. The concentrations of dopant, copolymer, and polymer alloy, as well as the conditions for preparing HTL, ETL, and perovskite film, were fine-tuned carefully, and only the preparation conditions to obtain the highest efficiency cells (from the experiments carried out in this study) are reported in this paper.

Spiro-OMeTAD HTL was prepared from a 6.7 wt % spiro-OMeTAD chlorobenzene solution (containing 80 mg of spiro-OMeTAD, 28.5 μL of tBP, and 17.5 μL of 52 wt % Li-TFSI acetone solution in 1 mL of chlorobenzene) by spin coating. Thirty μL of spiro-OMeTAD solution was added on top of the perovskite film (the substrate is 1.5 cm × 1.5 cm) under the spin of 3000 rpm for 30 s and then exposed to air for 1 h before being sent to the vacuum system for coating with 5 nm MoO_3_ and a 100 nm Ag electrode.

### 4.3 Device Fabrication and Characterization

SnO_2_ ETLs (deposited on 1.5 cm × 1.5 cm patterned FTO electrode) were prepared as what we reported previously.^78^ The perovskite absorber was deposited on top of the ETL using a one-step, antisolvent dripping method from its precursor solution. The perovskite precursor solution was prepared by well mixing FAI, MAI, PbI_2_, and PbBr_2_ in DMF/DMSO (6:4, V/V) to form a 1.3 M (based on Pb) precursor solution with the stoichiometry of the perovskite as (FAI)_0.85_(PbI_2_)_0.89_(MAPbBr_3_)_0.15_ (or (FAI)_0.90_(PbI_2_)_0.94_(MABr)_0.10_(PbBr_2_)_0.10_).^79^ A 40 μL perovskite precursor solution was dripped on the ETL coated FTO with the spin program of 1000 rpm for 10 s, then 7000 rpm for 15 s, and at the last 3 s of the second spin rate, a 60 μL chlorobenzene was dripped on the wet film as an antisolvent. The resulting perovskite film (Psk) was heated at 150 °C for 10 min, then HTL was spin-coated on Psk followed by the vacuum deposition of 5 nm MoO_3_ as an electron-blocking layer and 100 nm Ag as an electrode. The processes for depositing the perovskite active layer were performed under a N_2_ filled glovebox. The coating of HTL on top of the Psk absorber, the thermal deposition of the MoO_3_ electron-blocking layer, and Ag electrode were described in the previous paragraph. The physicochemical properties and photovoltaic performance measurements of the PSC and its components were carried out using the same methods as reported previously.^78^ The efficiency of the cells was measured in a glovebox, but the long-term stability of the cells (nonencapsulated) was tested in the ambient atmosphere at 20 °C and 30% relative humidity.

## Supporting Information Available

Table S1, The photovoltaic performance of some HTLs using Li-TFSI and tBP or DPI-TPFB as a dopant; Table S2, The photovoltaic parameters of regular perovskite solar cells based on P15 and spiro-OMeTAD HTLs; Table S3, The carrier life-time of various HTLs deposited on perovskite films; Scheme S1, Procedure for the preparation of P15 copolymer; Figure S1, 1H-NMR spectra of acceptor, donor, and copolymer P15; Figure S2, (a) GPC and (b) TGA curves of P15; Figure S3, *I–V* curves of the regular perovskite solar cells based on seven HTLs; Figure S4, The distribution of the photovoltaic parameters of the cells based on five different HTLs; Figure S5, Water contact angles of various films; Figure S6, The Tauc plots (a) and UPS spectra (b) of various sample; Figure S7, The *I–V* curves under high applied voltage for the hole only devices based on P15, PDTON/P15, (P15 + PDTON), and PDTON/spiro-OMeTAD films(PDF)
