# Supplementary material for: Organic Salt-Doped Polymer Alloy: A New Prototype Hole Transporter for High-Photovoltaic-Performance Perovskite Solar Cells
Source: ACS Appl Mater Interfaces. 2025 Feb 5;17(7):10674–85. doi: 10.1021/acsami.4c19907 (PMC11843538; doi:10.1021/acsami.4c19907)
Supplement: Supplementary file 1 — am4c19907_si_001.pdf [file am4c19907_si_001.pdf]

Supporting Information

Organic salt doped polymer alloy: a new prototype hole transporter for high photovoltaic-performance perovskite solar cells

Bing-Chen Zhang, Shang-Wen Lan, Chia-Ha Tsai, Chien-Hung Chiang, Chun-Guey Wu*

Department of Chemistry, National Central University, Jhong-Li, 32001, Taiwan, ROC.

E-mail address of the corresponding author: Professor C.-G. Wu: t610002@cc.ncu.edu.tw

Table S1: The photovoltaic performance of some HTLs using Li-TFSI and tBP or DPI-TPFB as a dopant

**Absor-ber**	**HTL**	**dopant**	**Effici-ency**	**stability**	**Refer-ence**
Lead perovskite	spiro-OMeTAD	Li-TFSI and tBP	26.31%	stable	S(1)
Lead perovskite	spiro-OMeTAD	Li-TFSI and tBP	25.60%	stable	S(2)
Lead perovskite	spiro-OMeTAD	Li-TFSI and tBP	26.21%	stable	S(3)
Lead perovskite	spiro-OMeTAD	Li-TFSI and tBP	26.17	stable	S(4)
Lead perovskite	PTPD	DPI-TPFB	21.38%	stable	S(5)
Lead perovskite/Si tandem	PTAA	DPI-TPFB	27.8%	The encapsulated cell operated at their maximum power point for 200 h without loss of performance, and retaining ~83% of initial performance over a month of operation in an outdoor environment.	S(6)
Tin perovskite	spiro-OMeTAD	DPI-TPFB	10.9%	The encapsulated cell maintained 80% of its initial PCE under continuous light illumination for 1597 h at 45%–90% relative humidity.	S(7)
Lead perovskite	P15/ PDTON	DPI-TPFB	18.8%	The non-encapsulated cell maintained 60% of its initial PCE after exposing in air for 22 hours.	This work

Table S2: The photovoltaic parameters of regular perovskite solar cells based on P15 and spiro-OMeTAD HTLs.

**HTL**	**dopant**	**Jsc (mA/cm^2)**	**Voc (V)**	**FF (%)**	**Max PCE (%)**	**Average PCE (%)**
**P15**	none	23.6	1.0	50	11.76	10.89 ± 0.62
	LiTFSI, tBP	22.6	1.05	53	12.60	11.48 ± 0.76
	DPI-TPFB	24.6	1.03	60	15.08	13.57 ± 1.03
**Spiro-OMeTAD**	LiTFSI, tBP	25.3	1.13	73	20.9	20.01 ± 0.59

Table S3: The carrier life-time of various HTLs deposited on perovskite films

Sample for TRPL	τ_1 (ns)	A_1	τ_2 (ns)	A_2	τ_{avg} (ns)
Psk	1.70	56.9	9.53	43.1	5.07
Psk/P15	1.14	85.5	5.67	14.5	1.79
Psk/PDTON/P15	0.90	82.9	2.86	17.1	1.24
Psk/(P15+PDTON)	0.66	85.3	2.44	14.7	0.92
Psk/PDTON/ Spiro-OMeTAD	0.21	97.9	4.97	2.1	0.31

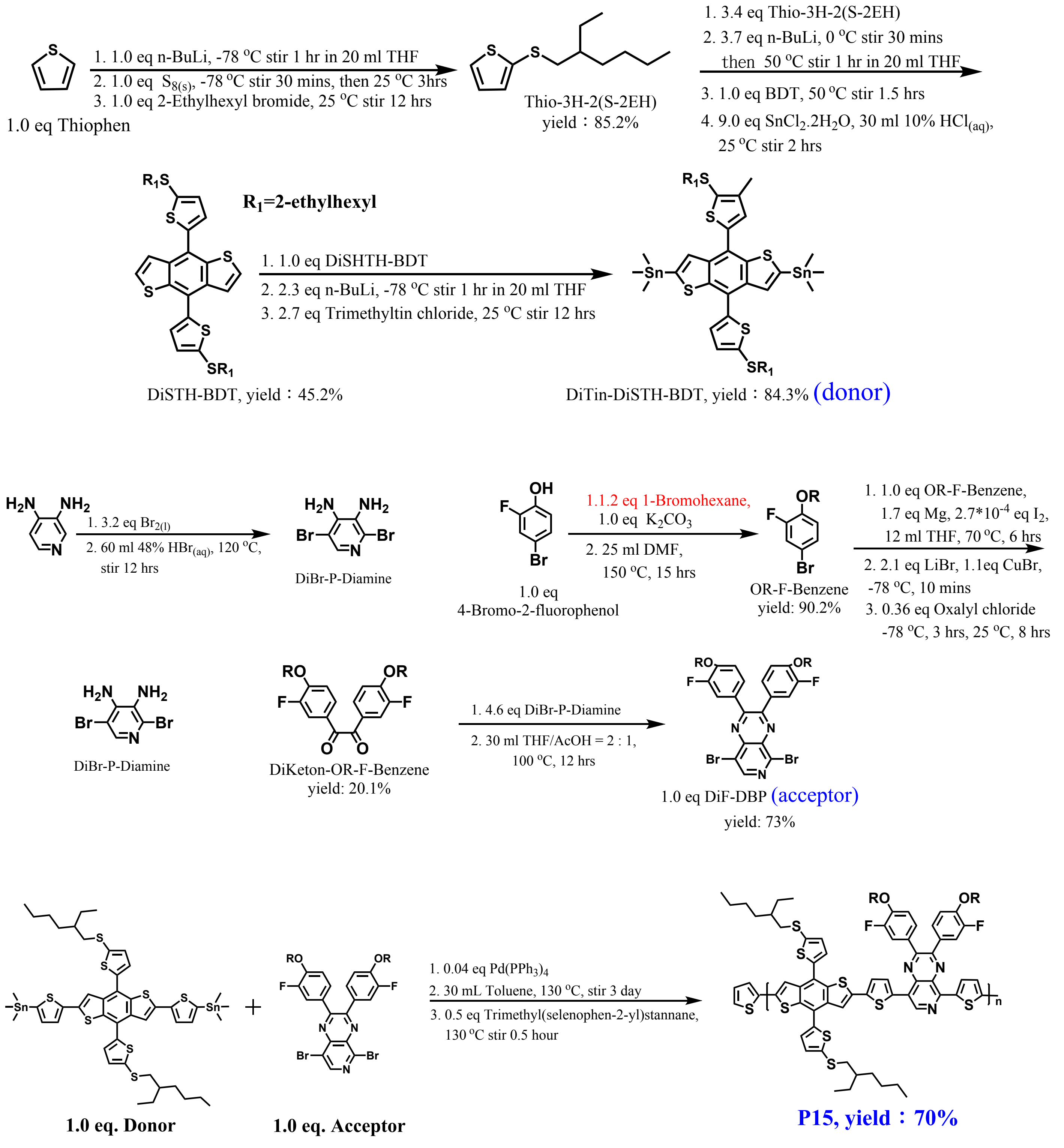

Scheme S1: Procedure for the preparation of **P15** copolymer.

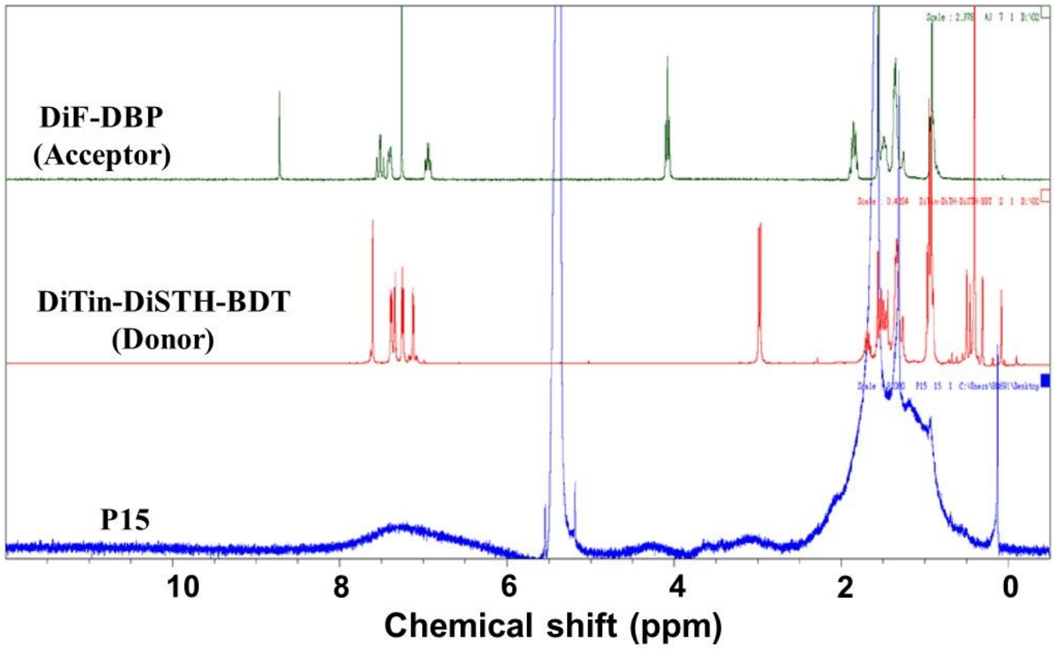

Figure S1: ^{1}H-NMR spectra of Acceptor, Donor, and copolymer **P15.**

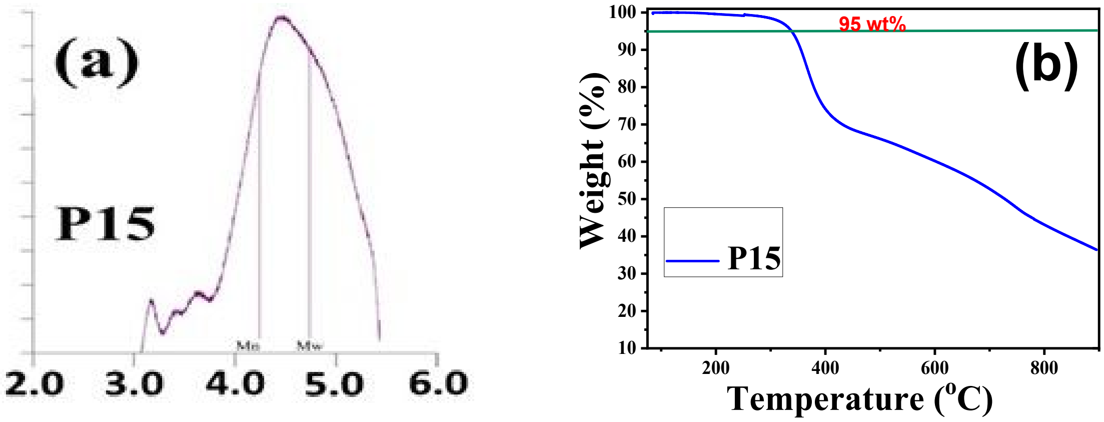

Polymer	Mn	Mw	Mp	PDI
P15	17548	55021	29832	3.13

Figure S2: (a) GPC and (b) TGA curves of **P15**.

(The data in the table under GPC curve were extracted from the GPC curve.)

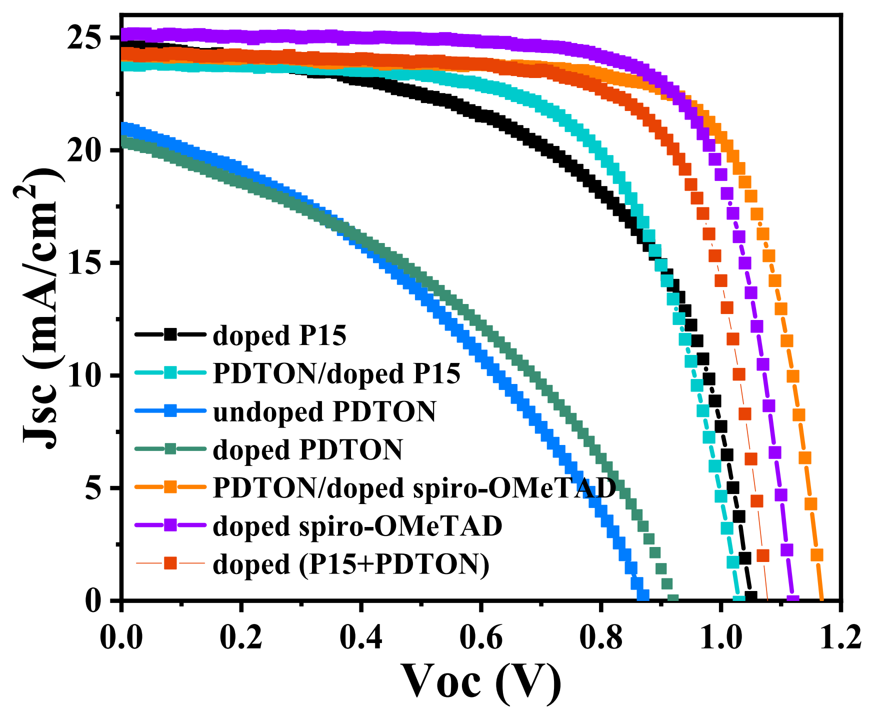

Figure S3: *I-V* curves of the regular perovskite solar cells based on seven HTLs.

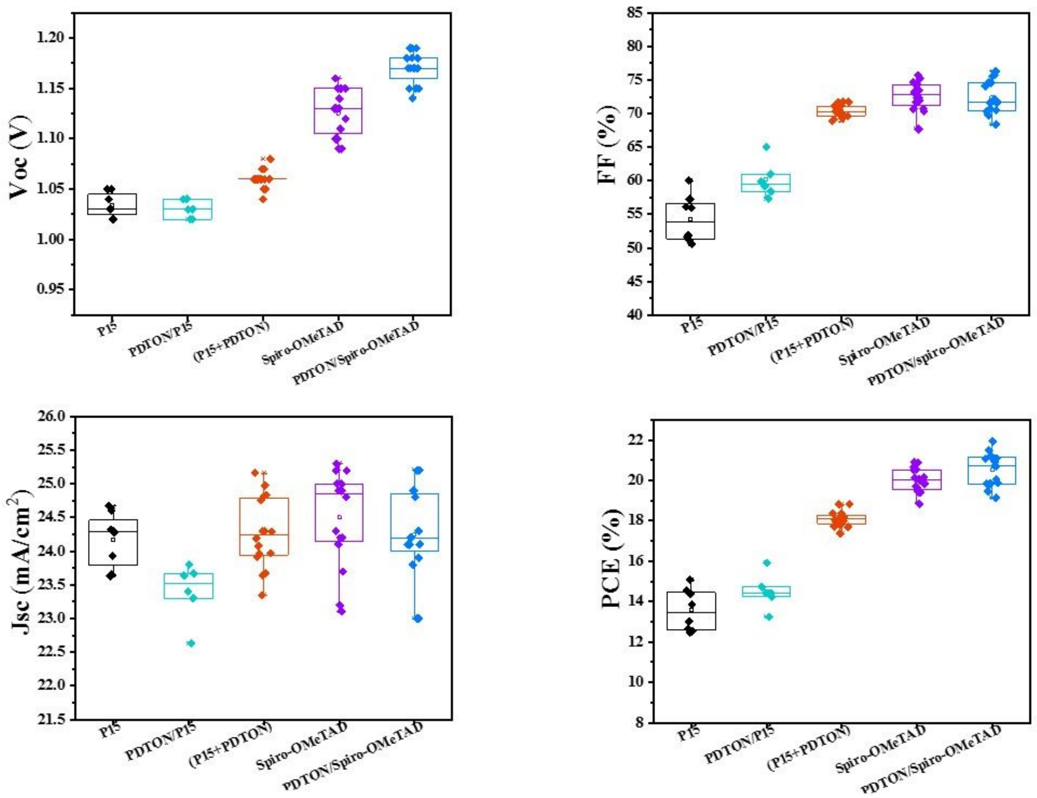

Figure S4: The distribution of the photovoltaic parameters of the cells based on five different HTLs.

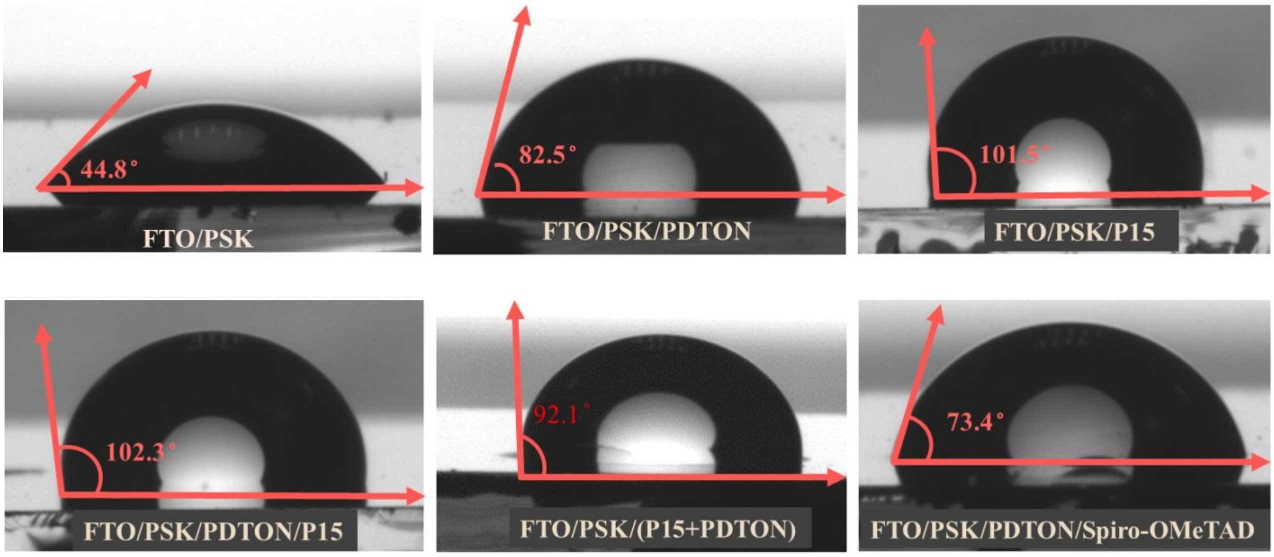

Figure S5: Water contact angles of various films.

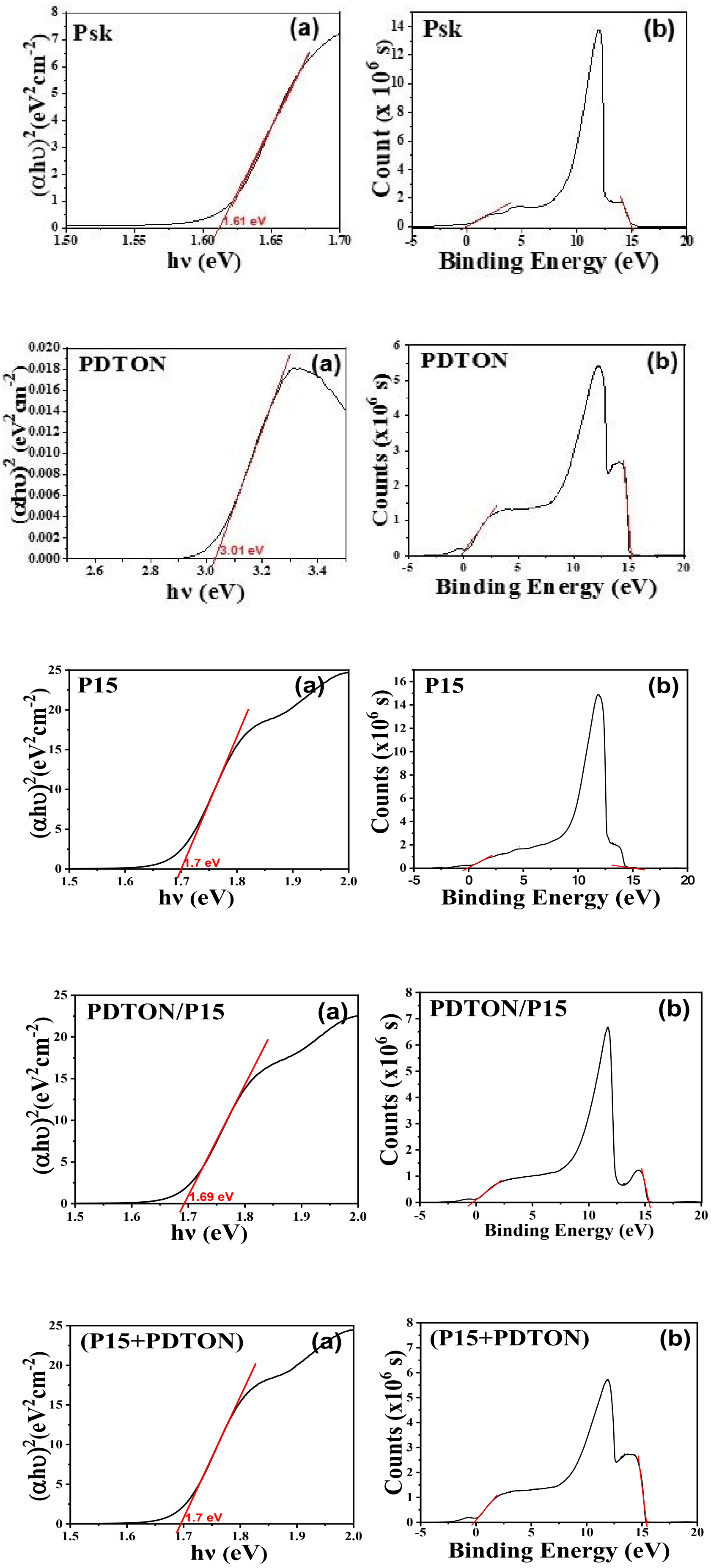

Figure S6: The Tauc plots (a) and UPS spectra (b) of various sample.

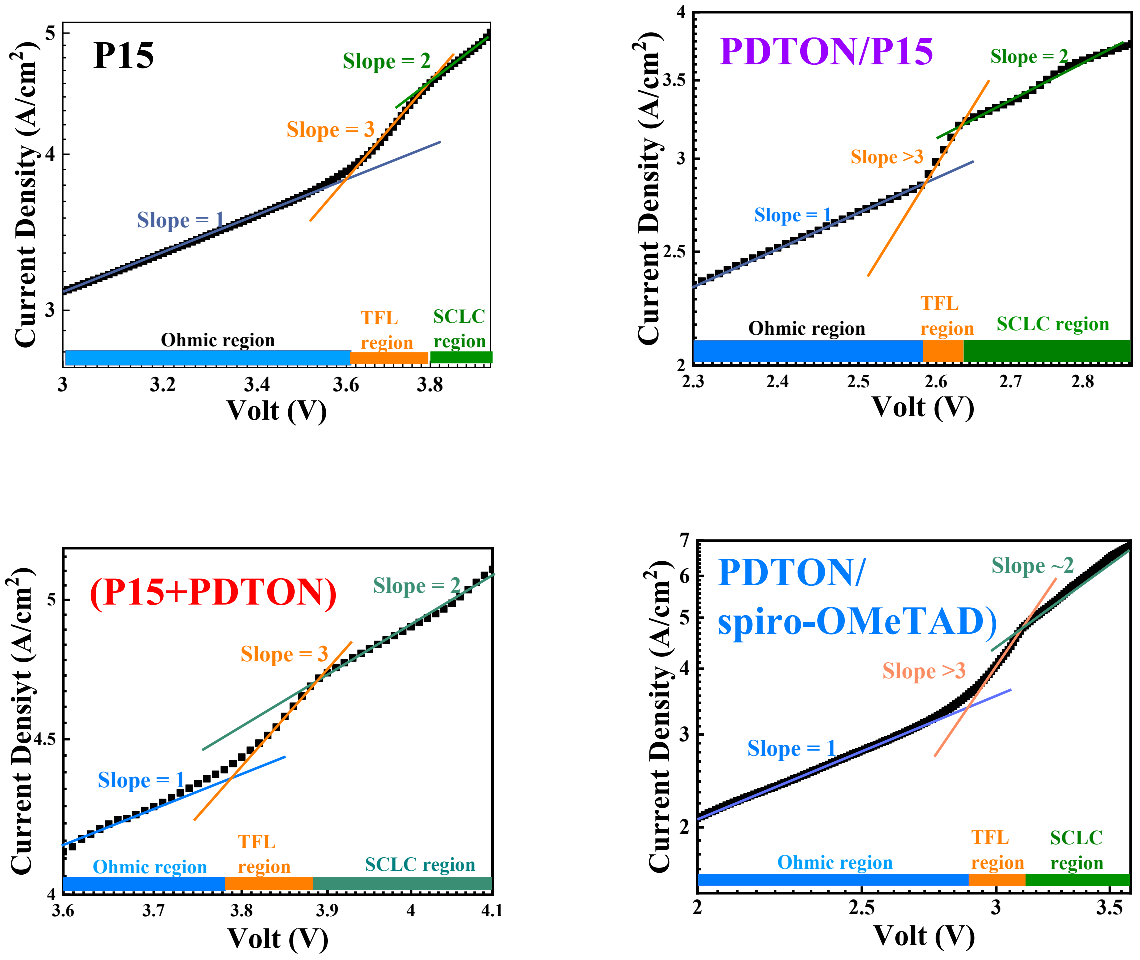

HTL	P15	PDTON/P15	(P15+PDTON)	PDTON/ Spiro-OMeTAD
**Mobility ($cm^2V^{-1}s^{-1}$)**	$1.36*10^{-4}$	$2.01*10^{-4}$	$3.39*10^{-4}$	$3.66*10^{-4}$

Figure S7: The *I-V* curves under high applied voltage for the hole only devices based on **P15,** PDTON/**P15**, (**P15**+PDTON), and PDTON/spiro-OMeTAD films. Data in Table under the *I-V* curves are the mobility data calculated from the *I-V* curves based on SCLC theory.

References:

S(1) Li, D.; Liu, H.; Hou, C.-H.; Yan, H.; Li, S.; Chen, P.; H.; Yu,W.-Y.; Zhao,Y.; Sui,Y.; Zhong, Q.; Ji, Y.; Shyue, J.-J.; Jia, S.; Yang, B.; Tang, P.; Gong, Q.; Zhao, L.; Zhu, R. Harmonizing the bilateral bond strength of the interfacial molecule in perovskite solar cells. *Nat Energy* **2024**, *9*, 1506-1516.

S(2) Zhao, Y.; Ma, F.; Qu, Z.; Yu, S.; Shen, T.; Deng, H.-X.; Chu, X.; Peng, X.; Yuan, Y.; Zhang, X.; You, J. Inactive $(PbI_2)_2RbCl$ stabilizes perovskite films for efficient solar cells. *Science* **2022**, *377*, 531-534.

S(3) Zhou, J.; Tan, L.; Liu, Y.; Li, H.; Liu, X.; Li, M.; Wang, S.; Zhang, Y.; Jiang, C.; Hua, R.; Tress, W.; Meloni, S.; Yi, C. Highly efficient and stable perovskite solar cells via a multifunctional hole transporting material. *Joule* **2024**, *8*, 1691-1706.

S(4) Shen, L.; Song, P.; Jiang, K.; Zheng, L.; Qiu, J.; Li, F.; Huang, Y.; Yang, J.; Tian, C.; Jen, A. K.-Y.; Xie, L.; Wei, Z. Ultrathin polymer membrane for improved hole extraction and ion blocking in perovskite solar cells. *Nat. Commun.* **2024**, *15*, 10908

S(5) Zhang, H.; Hui, W.; Wang, Z.; Li, M.; Xi, H.; Zheng, Y.; Liu, X. Ultraviolet-light Induced H^+ Doping in Polymer Hole Transport Material for Highly Efficient Perovskite Solar Cells. *Mater. Today Energy* **2022**, *30*, 101159.

S(6) Hu, M.; Risqi, A. M.; Wu, J.; Chen, L.; Park, J.; Lee, S.-U.; Yun, H.-S.; Park, B.-W.; Brabec, C. J.; Seok, S. I. Highly Stable n–i–p Structured Formamidinium Tin Triiodide Solar Cells through the Stabilization of Surface Sn Cations. *Adv. Funct. Mater.* **2023**, 2300693.

S(7) Zheng, X.; Liu, J.; Liu, T.; Aydin, E.; Chen, M.; Yan, W.; De Bastiani, M.; Allen, T. G.; Yuan, S.; R. Kirmani, A. R.; Baustert, K. N.; Salvador, M. F.; Turedi, B.; Alsalloum, A. Y.; Almasabi, K.; Kotsovos, K.; Gereige, I.; Liao, L.-S.; Luther, J. M.; Graham, K. R.; Mohammed, O. F.; De Wolf, S.; M. Bakr, O. M. Photoactivated p-Doping of OrganicInterlayer Enables Efficient Perovskite/SiliconTandem Solar Cells. *ACS Energy Lett.* **2022**, *7*, 1987-1993.
